# Injustice to transsexual women in a hetero-normative healthcare system

**DOI:** 10.4102/phcfm.v6i1.574

**Published:** 2014-11-21

**Authors:** Douglas Newman-Valentine, Sinegugu Duma

**Affiliations:** 1 Division of Nursing and Midwifery, University of Cape Town, South Africa

## Abstract

**Background:**

Transsexual women who are on the journey of sexual re-alignment will experience various health problems. These problems are related directly to the treatment regime that they are following in order to attain and maintain their physical embodiment as a woman. They are forced to negotiate a hetero-normative healthcare system in order to receive assistance and care for their health problems related to their sexual re-alignment process.

**Aim:**

The questions posed were: What are the unique health problems that transsexual women experience whilst on the journey of sexual re-alignment? What is the current context of the South African healthcare system in which transsexual women should negotiate healthcare? These questions were asked in order to explore the health problems with which transsexual women are faced and to describe the hetero-normative healthcare system in South Africa.

**Method:**

An electronic literature search was executed via the EBSCO host with specific inclusion and exclusion criteria. The search words that were used were: Transsexual/s and Health/Healthcare. All studies had to be peer reviewed and published in the English language, from January 1972 up until February 2013. Literature on transsexual children was excluded.

**Results:**

Transsexual women have the potential to suffer significant side-effects from their sexual re-alignment treatment, including cardio-vascular problems, endocrine problems and mental ill-health. They are also vulnerable to HIV infection. They have poor access to quality holistic healthcare and this may lead an increase in the mortality and morbidity figures of women.

**Conclusion:**

A hetero-normative healthcare system has a negative impact on the health of transsexual women and will cause them to be marginalised. This could contribute to both homo- and trans-phobia that will in turn strengthen the belief that transsexual women are un-African.

## Abstrait

**Injustice envers les femmes transsexuelles dans un système de santé aux normes hétérosexuelles.**

**Contexte:**

Les femmes transsexuelles qui sont sur le chemin du réalignement sexuel souffriront de différents problèmes de santé. Ces problèmes proviennent directement du traitement qu'elles suivent pour atteindre et maintenir leur corps de femme. Elles doivent surmonter un système de santé aux normes hétérosexuelles pour recevoir de l'aide et des soins pour leurs problèmes de santé liés au processus de réalignement sexuel.

**Objectif:**

Les questions posées étaient: Quels sont les problèmes de santé particuliers que les femmes transsexuelles rencontrent sur le chemin du réalignement sexuel? Quel est le contexte actuel du système de santé sud-africain avec lequel les femmes transsexuelles doivent négocier leurs soins? On a posé ces questions pour examiner les problèmes de santé auxquels les femmes transsexuelles sont confrontées et décrire le système de santé hétéro-normatif en Afrique du Sud.

**Méthode:**

On a effectué une recherche de littérature électronique par l'hôte EBSCO avec des critères spécifiques d'inclusion et d'exclusion. Les mots de recherche utilisés sont: Transsexuel/s et Santé/Services de santé. Toutes les études ont été vérifiées par les pairs et publiées en anglais, de janvier 1972 à février 2013. On a exclu la littérature sur les enfants transsexuels.

**Résultats:**

Les femmes transsexuelles peuvent ressentir des effets secondaires significatifs de leur traitement de réalignement sexuel, tels que des problèmes cardio-vasculaires, endocrines et de santé mentale. Elles sont aussi vulnérables à l'infection par le VIH. Elles n'ont pas accès à des soins de santé de qualité ce qui peut augmenter les chiffres de mortalité et de morbidité des femmes.

**Conclusion:**

Un système de santé hétéro-normatif a un impact négatif sur la santé des femmes transsexuelles et les amènera à être marginalisées. Cela pourra contribuer à l'homo- et trans-phobie qui à leur tour renforceront la croyance que les femmes transsexuelles sont contraires à la mentalité africaine.

## Introduction

Transsexual women experience significant health problems which are related directly to their gender re-alignment journey. They have to negotiate care in a hetero-normative healthcare system, which is exclusive.

The reviewed literature in this article explores the health problems which transsexual women experience and makes a description of the hetero-normative health care system in South Africa, after which appropriate recommendations for practice and research are made.

### Transsexual woman: A definition

The term Transsexual describes someone who identifies psychologically and emotionally as having a gender identity in opposition to that which was assigned at birth. Someone who identifies as transsexual or transgender may want to make changes to their body using hormones or gender confirming surgeries to transition their physical features to match their internal sense of gender.^1^

A transsexual individual can further be described as a person who wishes to be of the opposite sex and is in the process of gender re-alignment.^2^

A transsexual woman is a person who was sexually defined and registered as male at birth, but identifies psychologically as female. She will seek medical help to align her physical embodiment with her gender and will be placed on a regime of lifelong hormonal therapy and various gender-confirming surgeries.

### Hetero-normativity explained

Hetero-normativity can be described as the belief that heterosexuality is the ideal and that all other sexualities are inferior. Legal and social structures are thus designed to accommodate heterosexuality, thus denying rights and status to different sexualities.^3^

The phenomenon of hetero-normativity is evident in all sectors of daily life. In most of the African countries, marriage is only recognised between a man and a woman. This immediately gives a superior status to the heterosexual union. At government institutions, banks, schools and universities, one of the first categories in which people are placed is either male or female. Even in culture, hetero-normativity is so deeply ingrained that it is often missed. When a child is born, the first information that a midwife or doctor will give a mother is the sex of the child. Family will usually enquire about the sex of a child before they question the health and well-being of the mother and the baby. This will be followed by ‘gender appropriate’ gifts and toys, as people naturally see gender and sex as being interchangeable.

## Methods

The literature review was done in a systematic manner. The search for the literature was done electronically via the EBSCO host with specific inclusion and exclusion criteria. The search words that were used were: Transsexual/s and Health/Healthcare. All studies had to be peer reviewed and published in the English language, anywhere from January 1972 up until February 2013.

All studies that were published in the Cinhal, Health Source, Humanities International Complete, Medline, Psyc Articles and Soc Index databases and which were found to be relevant to transsexuals and healthcare were included in the study. All studies that were specific to transsexual children under the age of 18 were automatically excluded.

The initial search yielded 172 studies. A total of 105 of the studies were excluded because of both the inclusion and exclusion criteria and repetition of studies on the databases. This exclusion process yielded 67 studies that were selected for the critical appraisal process.

A CASP (Critical Appraisal Skills Programme) critical appraisal tool was utilised to measure the quality of the 67 studies that were selected.^4^ After this process, another 37 studies were excluded because of insufficient rigour or reliability, leaving 23 studies which were finally selected for review.

Appropriate grey literature, such as acts and policies, was also included in this article.

## Review findings

### The South African healthcare system in context

Chapter two of the *Constitution of the Republic of South Africa* proclaims that healthcare is a human right for all citizens. It states that each citizen has a right to healthcare services, including reproductive healthcare.^5^ Furthermore, the *National Health Act* 2004 aims to provide the best possible health services to citizens. It is written that it protects and promotes the rights of vulnerable groups, including women, children, older persons and the disabled.^6^

Both the *Constitution* and the *National Health Act* are specific regarding who exactly would be defined as a vulnerable group, however, transsexual women are get specified. The National Department of Health has initiated and implemented various strategies in order to improve the health status of the South African population, but all of these initiatives are silent with regard to transsexual women.

The latest reform in the South African healthcare system is the National Health Insurance (NHI). The main objectives for the implementation of the NHI in South Africa are to bring reform, improve service and to promote equity and efficiency in the healthcare system.^7^ Throughout this document, the issue of equity is discussed in detail, yet transsexual women seem to be excluded in this latest plan of the South African government.

In the South African healthcare system, a person is treated as either male or female and there are various health programmes that place the focus of care on either men or women, such as the women's health and men's health programmes. This rigid form of gender identification within the health system will place people into two distinct boxes of care, with no space for people who are identified as different. With the definition of hetero-normativity in mind, where heterosexuality is seen as the norm, the South African healthcare system in effect treats people strictly as heterosexual men and/or women.

Hetero-normativity is a form of discrimination that is present in the healthcare system of South Africa, but it is not accepted as such. This leads to an exclusive healthcare system which denies a subset of the population, such as transsexual women, access to quality appropriate healthcare.

The silence with regard to the protection of sexual minority groups in the healthcare system might result in an increase in the morbidity and mortality figures of the country and is in direct conflict with the aims and objectives of the health legislation of South Africa.

### Health problems of transsexual women

Literature shows that the life-long administration of feminising hormones used by transsexual women has the potential to lead to an increase in both systolic and diastolic blood pressure. Furthermore, it can increase insulin resistance in transsexual women.^8^ Pulmonary embolism, deep venous thrombosis and osteoporosis may also occur.^9^ Transsexual women require chronic management to treat conditions such as diabetes and hypertension. What makes these chronic conditions unique to transsexual women, is that they are subjected to a regimen of lifelong hormonal treatment in order to facilitate and maintain their transitioning and sexual re-alignment.

The chronic infection, HIV, may also affect transsexual women. Two large studies, conducted in the United States of America (USA), identified that 40% of the participants reported inconsistent condom use. Less than half of the respondents made use of protection during their last sexual encounter, whilst 64% of the respondents reported having engaged in high-risk sexual activity during the past three months.^10, 11^

Transsexual women are anatomically more vulnerable to HIV because of the vulnerability of the tissue in the neo-vagina and the rectal mucosa.^11^ The immediate question asked would be why this subset of the community would take such health risks knowing that they might become infected with either HIV or other sexually-transmitted infections (STIs). It is not clear if their attending health providers inform these women about the dangers of contracting HIV and other STIs.

In 2002, Kenagy found, in a needs assessment survey, that male-to-female (MTF) transsexual women believe that they will not become infected with HIV and thus indulge in risky behaviour.^12^ HIV prevention programmes in South Africa target groups other than transsexual women. It is possible that the absence of health education for this group perpetuates the belief on the part of transsexual women that they are not at risk for HIV infection.

Two USA-based qualitative studies concluded that transsexual women have a need to be accepted. They participate in high-risk sexual encounters in order to avoid rejection.^13^

Two surveys conducted in Rome and California, reported that 20% and 52%, respectively, of the transsexual women surveyed were HIV positive. It was noted that this was possibly an underestimation, since the respondents self-reported their HIV status.^14, 15^

South Africa has a female HIV prevalence rate of approximately 17.4%,^16^ which is high in comparison with international statistics. This elevated prevalence of HIV amongst women is a concern and could suggest an even higher infection rate amongst transsexual women because of their vulnerability.

Apart from their physical healthcare needs, transsexual women are at great risk of mental ill-health as a result of the dramatic changes they experience during their transitioning process. A USA-based survey, which looked at 446 transsexual individuals, found that transsexual women have diminished mental health as opposed to their heterosexual counterparts.^17^ In 2010, Hoshiai and others, identified that 72% of transsexual women had suicidal ideation, which can be correlated directly with the poor state of their mental wellness. Furthermore, androgen deprivation, which forms an integral part of the treatment toward gender re-alignment, can lead to depression.^18^

The prevalence of homophobia against Lesbian, Gay, Bi-Sexual, Transgendered and Intersex (LGBTI) youth is high^19^ and it is recognised that a higher suicide risk is present in this group.^20^ It can be concluded that transsexual women may potentially be at higher risk for suicide because they are even more marginalised than other people of the Lesbian, Gay, Bi-Sexual (LGB) community and often suffer internalised stigma.^21^

From the above, it is clear that supporting the mental well-being of this subset of the community is challenging. It is exacerbated by homo- and trans-phobia, stigma and depression, possibly caused by androgen deprivation.

The available literature demonstrates that transsexual women are at risk of the development of hypertension, diabetes, embolism, osteoporosis, HIV and mental illness amongst others. These conditions are directly related to their journey of sexual transition and will require them to seek appropriate healthcare at all available levels of care.

### Access to appropriate healthcare services

Literature from North and South America shows that access to available services for the LGBTI community remains largely limited, or that there is a complete absence of healthcare services available to the transsexual community.^22, 23^ In the USA, both African-American and uninsured, impoverished, foreign-born transsexual women, are reported to have the least access to healthcare services.^22, 24^

Adequate access to healthcare and provision of hormone therapy significantly increase the quality of life of transsexual women. Of critical importance is the availability of trans-specific health education, which could have a positive influence on their health risk behaviour patterns.^16, 25^ The knowledge of healthcare providers regarding transsexual health issues is vital for their access to and utilisation of appropriate healthcare services.

In the USA, a study of 101 MTF transsexual women found that a barrier to effective healthcare is the limited access to a healthcare provider that has adequate knowledge about the specifics of transsexual health.^26^

Another smaller study showed that specific health programmes do not acknowledge the LGBTI community,^19, 20^ but in a patient satisfaction survey of 180 transsexual women, it was confirmed that transsexual women consider their healthcare needs to be important.^27^

Health professionals’ attitude, such as respect and sensitive care, is another critical factor toward increasing access and utilisation of healthcare services by the transsexual women. This was highlighted in a study where it was found that transsexual women from Australia, America and Europe choose to have trans-related procedures performed in Thailand. The Thai healthcare system is more accepting of the needs of transsexual women.^28^

Evidence suggests that having adequate access to trans-related healthcare is directly proportional to positive health outcomes for transsexual women. An improvement in the quality of healthcare provided to transsexual women will have a positive impact on the morbidity and mortality statistics of this group.

### Transsexual women in Africa

Research in Africa is silent with regard to access to healthcare for transsexual women. It is assumed that the reason for this silence is because of the fact that any sexuality other than heterosexuality is deemed illegal in the significant majority of African countries and Africa has a very strong hetero-normative culture. It is then further assumed that African transsexual women will have poor access to quality healthcare and will contribute to the already high mortality and morbidity figures.

The population of transsexual women in South Africa is unknown. Hetero-normative census data does not make provision for documenting a transsexual woman as such. Through working with transsexual women, it has been observed that the community of transsexual women is fluid in nature. They will often move to protect their safety as they are under constant threat of transphobic violence, including rape. The violence and rape are a result of the society's hetero-normative belief and people will condone the violence and rape of these women because they are seen as lesser beings that can be treated in any manner.

With little known about the population of transsexual women, transsexual women indeed contribute to the mortality and morbidity figures of women. International literature clearly states that these women will experience health problems that may lead to death and chronic illness if not managed appropriately.

The difficulty arises in the reporting of mortality and morbidity figures of transsexual women, as there is no provision made in the hetero-normative reporting system for the reporting of this data. Even after death, transsexual women remain silent in the healthcare system. Without adequate reporting systems, audits are impossible and no evidence will be available to influence positive change in healthcare services for these women.

## Conclusion

Literature suggests that transsexual women could potentially suffer significant chronic side-effects as a result of their treatment, such as diabetes, hypertension, osteoporosis and cancer, amongst others. These side-effects could be managed successfully should health practitioners have enough knowledge regarding trans-related healthcare. It is recommended that health practitioners be trained to manage these clients who would present at primary healthcare clinics with their trans-related health problems. This will ensure holistic care for these women.

Transsexual women are silent in health reporting systems. It is recommended that transsexual women should be noted as such on admission to any healthcare institution and should form part of data collected in healthcare systems.

Trans-phobia is a common phenomenon. This might lead to violence in the form of physical and sexual abuse of transsexual women. These violations should be noted as ‘Transphobic violence’ in order to have focused treatment and to influence prevention campaigns.

Amongst various factors, the vulnerability of the neo-vagina of transsexual women and the susceptibility of the rectal mucosa will make them vulnerable to the contraction of HIV. It is recommended that health education programmes that place a focus on the prevention of HIV should include transsexual women. This could increase awareness amongst transsexual women themselves and could also inform the broader population regarding the existence of transsexual women. It is also recommended that transsexual women be included in all other health education programmes in order to reduce stigma.

Research needs to be done to describe transsexual women in Africa. Their health problems should be investigated and reported. The phenomenon of homophobic violence, which includes rape, should also be investigated and reported as such.

These strategies will give a voice to an otherwise marginalised people, it will create awareness of the problems that a silenced community experience and it may start to break the stigma that any sexuality other than heterosexuality is un-African.
